# Distributional Learning of Lexical Tones: A Comparison of Attended vs. Unattended Listening

**DOI:** 10.1371/journal.pone.0133446

**Published:** 2015-07-27

**Authors:** Jia Hoong Ong, Denis Burnham, Paola Escudero

**Affiliations:** The MARCS Institute, University of Western Sydney, Sydney, New South Wales, Australia; Max Planck Institute for Human Cognitive and Brain Sciences, GERMANY

## Abstract

This study examines whether non-tone language listeners can acquire lexical tone categories distributionally and whether attention in the training phase modulates the effect of distributional learning. Native Australian English listeners were trained on a Thai lexical tone minimal pair and their performance was assessed using a discrimination task before and after training. During Training, participants either heard a Unimodal distribution that would induce a single central category, which should hinder their discrimination of that minimal pair, or a Bimodal distribution that would induce two separate categories that should facilitate their discrimination. The participants either heard the distribution passively (Experiments 1A and 1B) or performed a cover task during training designed to encourage auditory attention to the entire distribution (Experiment 2). In passive listening (Experiments 1A and 1B), results indicated no effect of distributional learning: the Bimodal group did not outperform the Unimodal group in discriminating the Thai tone minimal pairs. Moreover, both Unimodal and Bimodal groups improved above chance on most test aspects from Pretest to Posttest. However, when participants’ auditory attention was encouraged using the cover task (Experiment 2), distributional learning was found: the Bimodal group outperformed the Unimodal group on a novel test syllable minimal pair at Posttest relative to at Pretest. Furthermore, the Bimodal group showed above-chance improvement from Pretest to Posttest on three test aspects, while the Unimodal group only showed above-chance improvement on one test aspect. These results suggest that non-tone language listeners are able to learn lexical tones distributionally but only when auditory attention is encouraged in the acquisition phase. This implies that distributional learning of lexical tones is more readily induced when participants attend carefully during training, presumably because they are better able to compute the relevant statistics of the distribution.

## Introduction

How do we learn the regularities that exist in our highly structured environment? One approach is that we learn by tracking the statistics—ranging from simple frequency counts to complex conditional probabilities—of the regularities in the environment. This form of acquisition is broadly termed statistical learning and evidence shows that it is a domain-general mechanism which can be used to acquire auditory (linguistic and musical) and visual knowledge [1–3]. Distributional learning, in which the statistics tracked by the learner are merely the frequency count of the to-be-learned distribution [4], is a specific form of statistical learning which has been proposed to account for how phonetic categories are acquired. For example, Japanese listeners learn from their linguistic environment that there is only one category (unimodal or single peak distribution) along a particular acoustic dimension in Japanese, the Japanese /r/, whereas English listeners learn that there are two categories (bimodal distribution) along the same acoustic dimension, English /r/ and /l/, resulting in the well-known difficulty native Japanese adults face in discriminating English /r/ and /l/ [5].

This form of phonetic category acquisition has been studied empirically with consonants [4,6–8] and vowels [9–12]. The procedure in a distributional learning experiment is typically as follows: all participants are trained on a continuum spanning a minimal pair (e.g. /d/-/t/). For one group, the frequency of the training tokens along the continuum follows a distribution that should promote a single central category (a unimodal distribution); for another group, the frequency follows a distribution that should induce two separate categories towards the end of the continuum (a bimodal distribution). Discrimination performance on the target minimal pair between the two distribution conditions is compared after training. Distributional learning is said to occur when discrimination of the minimal pair (such as the end tokens of the continuum) by those trained on the bimodal distribution improve significantly more than those trained on the unimodal distribution.

It has been proposed that distributional learning underlies perceptual attunement [13,14], in which young infants, who are previously universal speech perceivers, are better at discriminating items that are specific to their environment (and therefore, familiar) and worse at discriminating unfamiliar items. For example, infants are initially able to discriminate virtually all speech sounds in the world but by their first birthday, their perception has become tuned to just those that are relevant in their linguistic environment [15–18]. However, because distributional learning has mostly only been studied with consonants and vowels [7–9,11], it remains to be investigated whether distributional learning underpins perceptual attunement of lexical tones.

In lexical tone languages, a change in pitch signals a change in meaning of the lexical item. For example, in Mandarin, the word /ma/ when spoken in a high level tone (/ma55/) means ‘mother’, whereas when spoken in a dipping tone (/ma214/), it means ‘horse’ (lexical tones are represented using Chao values [19] in this paper. Chao values represent a relative scale of pitch height and pitch contour from 1 to 5, with 1 being the lowest in pitch and 5 being the highest). Although approximately 70% of the world’s languages use tones to signal a change in meaning [20], lexical tone is very much understudied compared to consonants and vowels. In tone languages, the linguistic status of lexical tones is interesting as, on the one hand, their function is similar to segments (consonants and vowels) in that they are phonemic and, just like segments, tones also undergo perceptual attunement by the first year of life [21–23]. On the other hand, lexical tones are structurally more like suprasegmentals, such as intonations [22,24], in that fundamental frequency (F0, the physical property of pitch) overlays the speech form in the production of tones (lexical tones over vowels and intonation over words/sentences).

If learners do acquire lexical tones distributionally, this would suggest that at least in terms of acquisition, tones are similar to segments (i.e., consonants and vowels). A handful of distributional learning studies have investigated lexical tones either with infants or adults [25,26], Taken together, their results seem to suggest that after 12 months of age, there is no effect of distributional learning of lexical tones. However, this may be due to the choice of stimuli; those previous studies used Mandarin Tone 55 (high level) and Tone 51 (falling) and tested non-tone language participants such as Dutch [25] and Australian English [26] participants. For these participants, Tone 51 may be relatively easy to discriminate as it is acoustically similar to the declarative intonation in Dutch [27] and English [28]. In other words, participants may have relied on their native language intonation to discriminate the Mandarin tone minimal pairs instead of forming lexical tone categories based on the training distribution. Therefore, one of the aims of the present study is to examine whether non-tone language listeners are able to form lexical tone categories distributionally when a difficult minimal pair of lexical tones is used.

Typically, distributional learning experiments involve passive listening [7,8] based on the assumption that learners discover the distribution structure by tracking the items that they hear. However, some researchers suggest that statistical learning is more effective when attention is given to the to-be-learned items [29,30]. Furthermore, some suggest that the effect seen in several distributional learning studies that utilised an enhanced bimodal distribution, that is, a bimodal distribution in which the relevant acoustic cue is artificially exaggerated [9,10], is due to a top-down modulation of attention to the acoustic cue that signals the contrast between a minimal pair, rather than a stimulus-driven effect of the distribution per se [31]. In other words, the authors argue that the exaggeration of the acoustic property in an enhanced distribution of a minimal pair resulted in the learners being made aware of the relevant acoustic cue that would then bootstrap them to discriminate the minimal pair.

Thus, it appears that two different types of attention may affect statistical/distributional learning: (i) a general attention to the acquisition phase that encourages learners to attend carefully throughout the entire training stimuli set, which would then allow the learners to compute the relevant statistics of the training stimuli; and (ii) a stimuli-specific attention that allows learners to pick up the relevant acoustic properties necessary for discrimination. The former type of attention is reminiscent of those used in electrophysiological studies that have typically found a larger event-related potential (ERP) response when participants actively attended to the auditory stimuli than when they listened to the stimuli passively [32–34]. It is argued that attending to the auditory stimuli enables the participants to pay attention to every stimulus in the training set, which is necessary for formulating a robust representation of the standard that is used as a comparison to the deviant stimuli [35]. It is this type of general attention that has not been manipulated in the distributional learning research paradigm, which motivated the second aim of the present study: would learners show better distributional learning when the learners’ attention to the listening task is potentiated during the acquisition phase? Accordingly, two types of distributional learning are examined: *unattended listening* (Experiments 1A and 1B) and *attended listening* (Experiment 2).

In addition, we also wish to investigate the generalisability of distributional learning. Previous research has shown mixed results in terms of whether adults are able to generalise to other consonants with features similar to those on which they were trained [6,7], but it appears that they are able to generalise vowels across different speakers [9–12]. In this regard, we tested participants with items on which they were trained (Trained) and on similar but unfamiliar items to the participants (Novel) in one of two dimensions: Syllable (i.e., a change in initial consonant: /k^h^a/ and /na/), or Speaker’s Gender (i.e., female and male), or both. It is hypothesised that if adults can learn lexical tones by tracking frequency distributions just as for consonants and vowels, then it is predicted that the Bimodal group will divide the continuum into two separate categories while the Unimodal group will perceive the training continuum as a single category. Consequently, when tested with the end tokens of the training continuum, we predict that not only will the Bimodal group show an improvement in discriminating both Trained and Novel lexical tones at Posttest relative to Pretest, but that the Unimodal group will show *no* improvement, or even, a decrease in discrimination performance. This should occur because following training, the former should be facilitated by the emergence of two separate categories while the latter should show an interference in performance due to the merging of the continuum to one single category. This twofold outcome of the same mechanism, that is, distributional learning is termed ‘the distributional learning effect’. Furthermore, we predict that a larger distributional learning effect will be observed following the attended listening task (Experiment 2) compared to the unattended listening task (Experiments 1A and 1B).

## Experiment 1A: Unattended Listening

### Method

#### Participants

Eighty native Australian English listeners who were Psychology undergraduates (68 females; age range 17–44; M_age_ = 21.09, SD_age_ = 5.70) participated. None spoke a tone language and all reported normal hearing. Twenty four participants reported having musical training; however, none had more than two years of musical experience (≤ 0.5 year = 6; 1 year = 7; 2 years = 11).

#### Ethics Statement

The University of Western Sydney Human Research Ethics Committee approved the study protocol. All participants were recruited from the University of Western Sydney. Participants were given an information sheet and they provided written informed consent prior to participating in the experiment. Written informed consent was obtained directly from the handful of participants who were 17-year-old undergraduates as per the protocol approved by the University of Western Sydney Human Research Ethics Committee (in Australia, it is common for 17 (going on 18) year olds to begin their first year of undergraduate degree). Participants were given course credit for their participation.

#### Test stimuli

Four native Thai speakers (2 females) produced the stimuli, which were two syllables /k^h^a/ and /na/, each produced with two different tones (Tone 33 and Tone 241) resulting in four different Thai words. The choice of this tone pair is motivated by a previous study that found it is the most difficult for non-tone language listeners to discriminate [36]. Each speaker produced multiple tokens of each sound. Minimal pairs were formed between two tones of the same syllable by the same speaker. Four target minimal pairs were used in this experiment as follows: Female 1 /k^h^a33/-/k^h^a241/; Female 2 /na33/-/na241/; Male /k^h^a33/-k^h^a241/; and Male 2 /na33/-/na241/ (see Table 1 for duration and F0 values over time). Having two different speakers of the same gender allows us to examine whether participants are able to generalise on a more abstract level rather than normalising to the speaker’s pitch range.

**Table 1:** Summary of the 24 test items used. Duration (in ms) and fundamental frequency (F0, in Hertz) at three normalised time points as well as the overall average of the tone space. Each speaker produced a minimal pair. Note that while duration differs across minimal pairs, the duration remains constant within each minimal pair.

Speaker/Syllable	Test Item			Tone Space F0 (Hertz)			
	Tone	Exemplar	Duration (ms)	10%	50%	100%	Mean
**Female 1 /k** ^**h**^ **a/**	33	1	680.35	271.09	257.74	256.04	261.59
	33	2	680.35	275.27	263.82	269.71	268.27
	33	3	680.35	273.78	269.63	269.77	271.11
	241	1	680.35	261.01	269.99	192.69	253.83
	241	2	680.35	269.04	268.31	174.29	247.61
	241	3	680.35	269.21	268.23	178.25	247.90
**Female 2 /na/**	33	1	831.85	214.31	210.75	200.32	209.59
	33	2	831.85	208.01	203.72	189.85	202.91
	33	3	831.85	217.04	211.89	207.29	212.67
	241	1	831.85	221.79	246.75	195.09	233.31
	241	2	831.85	234.93	239.75	185.27	229.75
	241	3	831.85	224.94	241.97	185.31	229.18
**Male 1 /k** ^**h**^ **a/**	33	1	493.38	147.70	142.86	136.39	142.04
	33	2	493.38	158.46	156.14	154.64	156.32
	33	3	493.38	168.05	166.03	164.63	166.02
	241	1	493.38	169.64	176.95	155.55	169.60
	241	2	493.38	161.38	160.12	119.96	148.94
	241	3	493.38	166.07	161.21	111.32	148.58
**Male 2 /na/**	33	1	731.23	115.19	110.52	104.17	109.93
	33	2	731.23	121.63	120.60	117.56	120.08
	33	3	731.23	121.81	119.02	117.03	119.38
	241	1	731.23	133.16	137.80	106.17	130.44
	241	2	731.23	127.55	122.32	91.74	116.39
	241	3	731.23	131.18	124.53	94.85	118.03

To ensure that only the pitch contour differed between the four target minimal pairs, we first chose a base waveform for each minimal pair that is comparable in duration and matching for the speaker. Then, we extracted the natural pitch contour from each member class of a minimal pair (Tone 33 and Tone 241) that were equivalent in duration and imposed it on the chosen base waveform for that particular minimal pair. Two further exemplars for each particular minimal pair were then generated by imposing the pitch contour of other natural recording tokens matching for the same word spoken by the same speaker on the same base waveform. Thus, within each minimal pair, there were three different exemplars for each tone, all of which have the same waveform pattern and differed only in their pitch contours. All the stimuli were normalised for amplitude (70dB). The duration of each minimal pair ranged from 493ms to 832ms, but the duration within each minimal pair was equal. The final stimulus set consisted of 24 tokens (2 words x 3 exemplars x 4 speakers), which were used as test stimuli (Table 1). The test stimuli formed a 2 x 2 factorial: Test Syllable (/k^h^a/ vs. /na/) x Test Gender (Female vs. Male speaker).

#### Training stimuli

**Fig 1:**
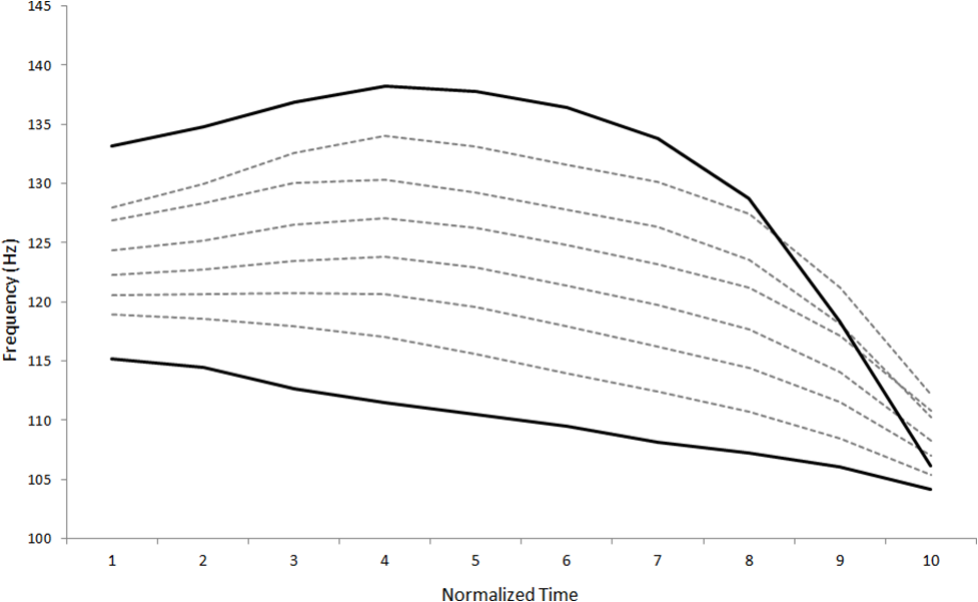
Pitch contour of the 8-step Male /na/ training continuum. Bold black lines represent the endpoints of the continuum (Token 1 and Token 8) while dashed grey lines represent the intermediate tokens (Token 2 to Token 7). Note that the pitch contours shown here represent the tone space of the vowel, in which the first 15% and the last 15% of the vowel were excluded to remove possible effects of coarticulation from the preceding consonant and creakiness, respectively.

To generate the training stimuli, we formed an 8-step continuum using Exemplar 1 of each minimal pair, with Tone 33 as Token 1 of the continuum and Tone 241 as Token 8. The continuum was created by interpolating the pitch contour of the two end tokens of each minimal pair (see Fig 1). This ensured that the pitch contour morphed from Tone 33 on one end of the continuum (Token 1) to Tone 241 on the other end of the continuum (Token 8), while keeping the waveform pattern consistent among the training tokens within each minimal pair. Both the test stimuli and training stimuli were presented to five native Thai listeners for verification. The test stimuli were identified correctly by the Thai speakers at least 80% of the time, and for each training continuum, there was a decline in percentage of Tone 33 response from Token 1 to Token 8, suggesting that the native listeners were sensitive to the change in pitch contour for the intermediate tokens [37].

#### Practice stimuli

A 440 Hz sinewave tone and a 440 Hz sawtooth tone, both 800ms in duration, were synthesized using Praat as practice stimuli for the ABX discrimination task.

#### Procedure

Participants were randomly assigned to one of two Distribution Conditions: Bimodal or Unimodal. Within each Distribution Condition, they were again randomly assigned to one of four training minimal pairs: Female /k^h^a33/-/k^h^a241/, Female /na33/-/na241/, Male /k^h^a33/-/k^h^a241/, or Male /na33/-/na241/. Thus, there were eight different conditions, each with 10 participants. The experiment was programmed on MATLAB 2012b and it was presented using an Acer TravelMate P653 laptop. The auditory stimuli were presented using a pair of Sennheiser HD650 headphones connected to an Edirol USB Audio Capture UA-25EX audio interface.

To familiarise the participants with the format of the ABX discrimination task, four practice trials were presented using the practice stimuli. The participants were told that they had to indicate whether the third sound, X, is similar to the first, A, or the second, B, by pressing the left shift key (A and X are similar) or the right shift key (B and X are similar). They were also informed that they only had 1s to respond in order to maintain vigilance.

There were three phases: Pretest, Training and Posttest. At Pretest and at Posttest, the participants were asked to discriminate all four test minimal pairs in an ABX discrimination task. In each trial, A and B were always Exemplar 1 tones, and X was either an Exemplar 2 or Exemplar 3 tone. For instance, a trial may consist of Female /kha33/_1_ - /kha241/_1_ - /kha33/_2_, where the subscripts indicate the exemplars. The four test minimal pairs were presented eight times each with the order of Exemplar 2 and Exemplar 3 tones as X being counterbalanced, resulting in a total of 32 trials in each test session, the order of which was randomised. There were no replacement trials for slow responses.

**Fig 2:**
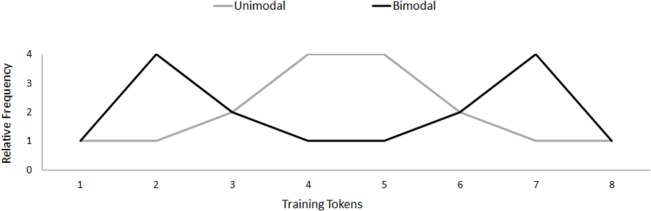
Unimodal and Bimodal distribution. For the Bimodal distribution, Token 2 and Token 7 were heard four times more often than the endpoints (Token 1 and Token 8), while for the Unimodal distribution, Token 4 and Token 5 were heard the most often relative to the endpoints.

During the training phase, the distribution of the training continuum was manipulated, depending on the Distribution Condition. As can be seen in Fig 2, the Bimodal participants heard Token 2 and Token 7 most frequently, whereas the Unimodal participants heard Token 4 and Token 5 most frequently. Crucially, the number of times both groups heard Token 1 and Token 8, that is, the A and B test stimuli, was the same. The training continuum was presented 16 times, that is, 256 tokens in total. While distributional learning experiments typically employ 128 training tokens [9,11,12], a pilot study for this experiment with 128 training tokens was conducted and we found no effect of distributional learning. Therefore, we doubled the number of training tokens in order to increase the chances of observing an effect. The order of the training tokens was randomised for each individual and the training phase took approximately five minutes in duration. Once the participants completed the experiment, they were given a language and musical background questionnaire. The entire experiment took approximately 30 minutes to complete.

### Results

**Fig 3:**
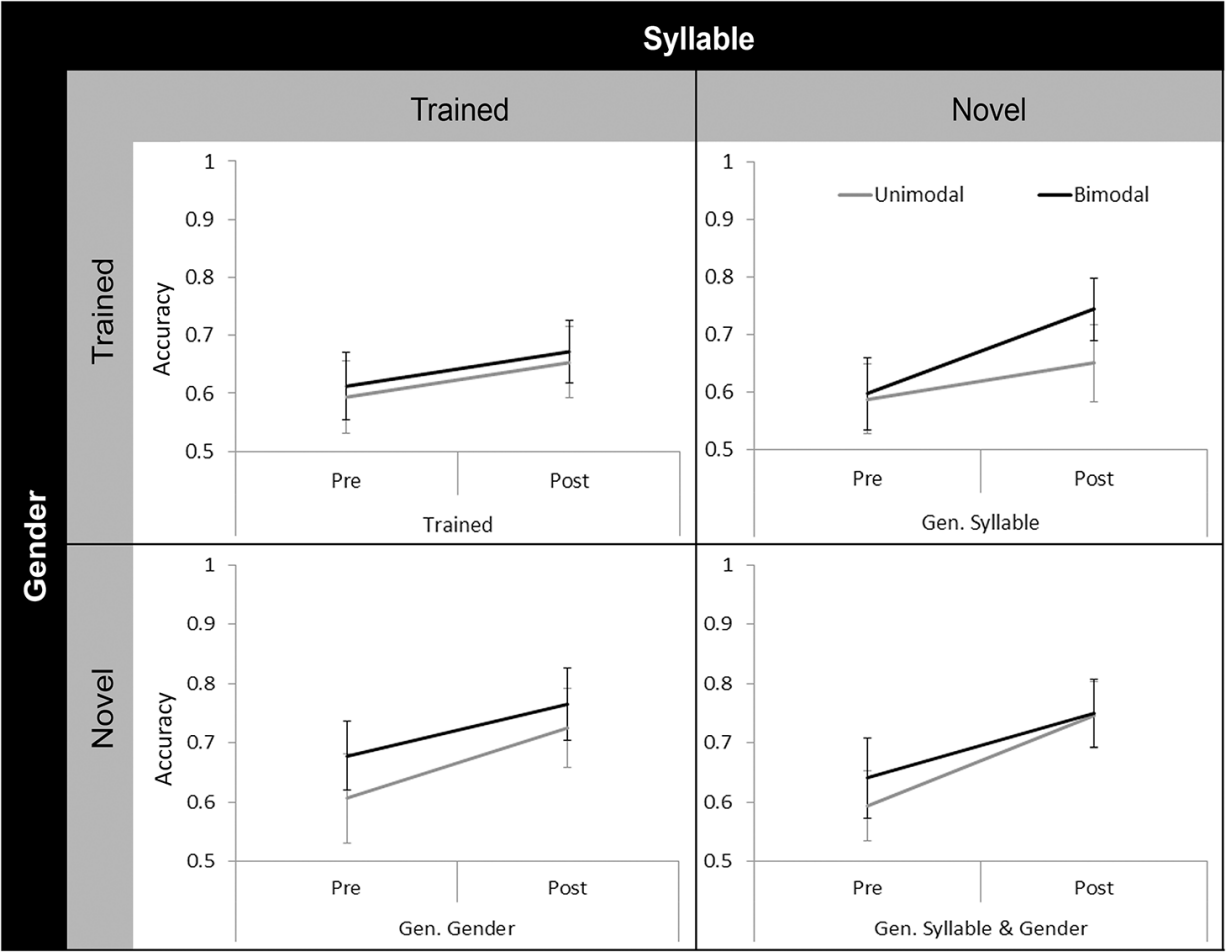
Comparison of Pretest and Posttest scores by Distribution Condition in Experiment 1A. Test items are coded based on what the participant was trained. For example, if a participant was trained on Male /na/ training continuum, then Trained represents Male /na/ test items and Generalised Syllable (Gen. Syllable) represents Male /k^h^a/ test items. In general, Posttest scores were higher than Pretest scores. Error bars represent 95% confidence intervals.

Firstly, a 2 (Distribution Condition) x 4 (Training Continuum) x [2 (Session) x 2 (Test Syllable) x 2 (Test Gender)] mixed ANOVA was conducted to determine whether there were any differences across training minimal pairs. Since there was no main effect of training minimal pairs (*F*(3, 72) = 2.063, *p* = .113, η_P_^2^ = .079), accuracy data were collapsed across training minimal pairs and the resulting data were analysed using a 2 x (2 x 2 x 2) Mixed ANOVA, with Distribution Condition (Unimodal vs. Bimodal) as a Between Subjects factor; and as Within Subjects factors: Session (Pretest vs. Posttest), Test Syllable (Trained vs. Novel) and Test Gender (Trained vs. Novel). Fig 3 shows the comparison of Pretest and Posttest scores on the four test items by Distribution Condition. There was a main effect of Session (*F*(1, 78) = 42.377, *p* < .001, η_P_^2^ = .352): Posttest scores (M = .713, SE = .015) were generally higher than Pretest scores (M = .614, SE = .015). Unexpectedly, there was also a main effect of Test Gender (*F*(1, 78) = 9.214, *p* = .003, η_P_^2^ = .106), suggesting that the participants were better at discriminating Novel Gender test items (M = .688, SE = .016) than Trained Gender test items (M = .639, SE = .014). The predicted Session by Distribution Condition interaction was not significant.

In order to determine whether the participants improved significantly from Pretest to Posttest on particular test dimensions, a set of one-sample *t*-tests was also conducted on difference scores (i.e., Posttest Scores – Pretest Scores) on Trained and Novel Syllable stimuli (collapsing across Speaker’s Gender), as well as Trained and Novel Gender stimuli (collapsing across Syllable) test items for both Unimodal and Bimodal conditions. For both Distribution Conditions, all one-sample *t*-tests revealed that the scores were significantly different from zero, except for the Trained Gender test items by the Unimodal group after Holm-Bonferroni correction (Unimodal Trained Gender, *t*(39) = 2.545, *p* = .015 vs. Unimodal: Trained Syllable, *t*(39) = 3.576, *p* = .001; Novel Syllable, *t*(39) = 3.601, *p* = .001; Novel Gender, *t*(39) = 5.029, *p* < .001. Bimodal: Trained Syllable, *t*(39) = 2.815, *p* = .008; Novel Syllable, *t*(39) = 4.176, *p* < .001; Trained Gender, *t*(39) = 3.794, *p* = .001; Novel Gender, *t*(39) = 3.159, *p* = .003).

### Discussion

The current experiment investigated whether non-tone language listeners are able to learn lexical tones distributionally after being trained on a passive listening task. The results showed across Distribution Conditions that generally there were higher discrimination scores at Posttest compared to Pretest. Contrary to previous distributional learning studies [4,6] in which improvement is found for Bimodal but not Unimodal exposure, the participants in both the Bimodal *and* Unimodal conditions in the present study performed above chance. In other words, while the Bimodal group improved following training as predicted, so did the Unimodal group, which prevents us from conclusively claiming that there is an effect of distributional learning. The discrepancy in results could be due to a difference in the experimental design or a difference in the nature of the stimuli. With respect to design, Maye and Gerken (4,6) employed a training-test phase design while the current study followed a pretest-training-posttest design [9]. Thus, the significant main effect of Session in the present study could simply reflect practice effects for the test stimuli. With respect to stimuli, Maye and Gerken (4,6) used consonants and here lexical tones were used. Further evidence relevant to these alternatives is provided in the following experiments.

The results indicate that the gender of speaker at test that was novel (i.e., Novel Gender) was easier to discriminate than the speaker’s gender at test on which the participants had been trained (i.e., Trained Gender). More relevant to our hypothesis, the results suggest that despite the improvement from Pretest to Posttest, there was no greater improvement for the Bimodal than for the Unimodal conditions and so, no effect of distributional learning of naturalistic lexical tones. This unexpected finding could be due to the fact that different groups were trained on different minimal pairs, which are not equivalent in initial difficulty. Indeed, a 2 (Test Syllable) x 2 (Test Gender) repeated ANOVA on Pretest accuracy scores revealed a main effect of Test Gender (*F*(1, 79) = 11.875, *p* = .001, η_P_^2^ = .131), suggesting that female stimuli were easier to discriminate than male stimuli. In addition, a Test Gender by Test Syllable two-way interaction (*F*(1, 79) = 14.324, *p* < .001, η_P_^2^ = .153) revealed that while there was no difference between the participants’ performance on the Female and Male /k^h^a/ test items, the participants performed significantly better on Female /na/ than on Male /na/ test items. Thus, the effect of distributional learning (if it exists) may have been masked by group differences in discrimination performance after being trained on a specific minimal pair. Furthermore, there were only 10 participants in each group. In order to address these concerns, we repeated the experiment with two changes: (i) increasing the number of participants, and (ii) training the participants on a single minimal pair instead of assigning the participants to one of four training minimal pair groups.

## Experiment 1B: Unattended Listening on Male /na/ Training Minimal Pair

Experiment 1B is similar to Experiment 1A except that more participants were tested (25 in each Distribution Condition) and only one training minimal pair was used: Male /na33/-/na241/. This choice is motivated by the fact that it is the most difficult to discriminate among the four minimal pairs, with the assumption that participants would benefit more from being trained on a difficult contrast [38].

### Method

#### Participants

Participants consisted of 50 native Australian English listeners (39 females), 20 of whom were a subset from Experiment 1A with 30 extra participants recruited for this experiment (15 extra participants in each Distribution Condition). The participants’ ages ranged between 17 and 40 years old, with an average age of 21.04 (SD_age_ = 5.52). None spoke a tone language and all reported normal hearing. Twelve participants reported having minimal musical training (≤ 0.5 year = 4; 1 year = 4; 2 years = 4).

#### Ethics Statement

The University of Western Sydney Human Research Ethics Committee approved the study protocol. All participants were recruited from the University of Western Sydney. Participants were given an information sheet and they provided written informed consent prior to participating in the experiment. Written informed consent was obtained directly from the handful of participants who were 17-year-old undergraduates as per the protocol approved by the University of Western Sydney Human Research Ethics Committee (in Australia, it is common for 17 (going on 18) year olds to begin their first year of undergraduate degree). Participants were given course credit for their participation.

#### Stimuli

The same stimuli as in Experiment 1A were used for the two testing sessions (Pretest and Posttest).

#### Procedure

The procedure was the same as Experiment 1A, with the exception that all the participants were trained on the Male /na33/-/na241/ training minimal pair.

### Results

**Fig 4:**
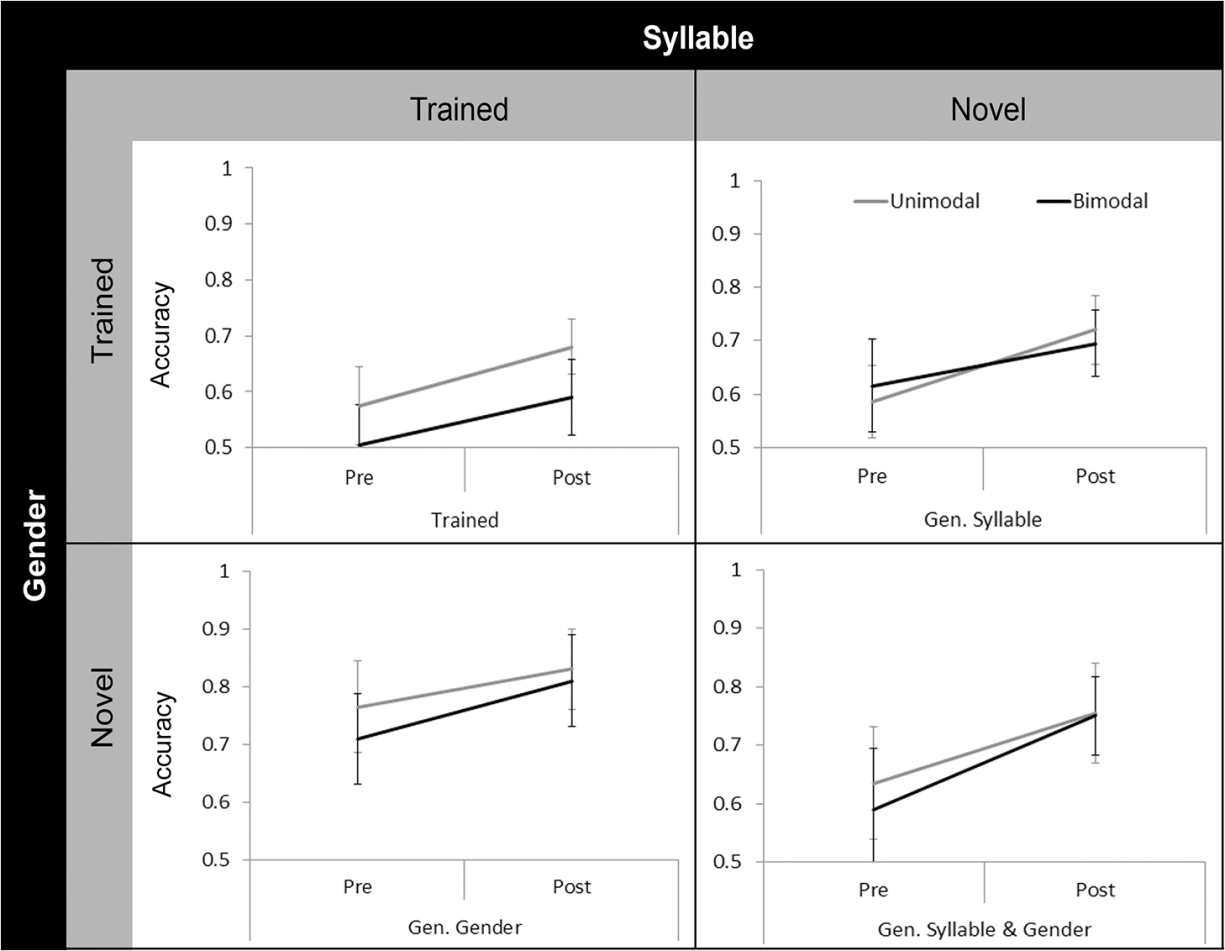
Comparison of Pretest and Posttest scores by Distribution Condition in Experiment 1B. Since only Male /na/ training continuum was used in this experiment, Trained, Gen. Syllable, Gen. Gender and Gen. Syllable & Gender test items represent scores on Male /na/, Male /k^h^a/, Female /na/ and Female /k^h^a/ test items, respectively. In general, Posttest scores were higher than Pretest scores. Error bars represent 95% confidence intervals.

The same analysis as in Experiment 1A was conducted in Experiment 1B: a 2 x (2 x 2 x 2) Mixed ANOVA with Distribution Condition (Unimodal vs. Bimodal) as a Between Subjects factor; and as Within Subjects factors: Session (Pretest vs. Posttest), Test Syllable (Trained vs. Novel) and Test Gender (Trained vs. Novel). Fig 4 illustrates the Pretest and Posttest scores on each test item by Distribution Condition. The ANOVA revealed a main effect of Session (*F*(1, 48) = 36.505, *p* < .001, η_P_^2^ = .432) with Posttest scores (M = .729, SE = .016) higher than Pretest scores (M = .623, SE = .020); and a main effect of Test Gender (*F*(1, 48) = 31.711, *p* < .001, η_P_^2^ = .398): Female stimuli (Novel Gender; M = .731, SE = .021) were easier for the participants to discriminate than Male stimuli (Trained Gender; M = .621, SE = .015). There was also a Test Gender by Test Syllable interaction (*F*(1, 48) = 20.791, *p* < .001, η_P_^2^ = .302). Simple main effects analysis revealed that the participants’ discrimination performance on Female and Male /k^h^a/ test items did not differ (Female Speaker: M = .683, SE = .026 vs. Male Speaker: M = .654, SE = .019), but their performance on Female /na/ test items (M = .779, SE = .022) was higher than that of Male /na/ test items (M = .588, SE = .019). Importantly, just like in Experiment 1A, there was no Session by Distribution Condition interaction.

A set of one-sample *t*-tests on difference scores was conducted to determine if participants in the two Distribution Conditions improved significantly from Pretest to Posttest. After the Holm-Bonferroni correction was applied, both groups still showed significant improvement on all test aspects from Pretest to Posttest (Unimodal: Trained Syllable, *t*(24) = 3.404, *p* = .002; Novel Syllable, *t*(24) = 3.579, *p* = .002; Trained Gender, *t*(24) = 5.018, *p* < .001; Novel Gender, *t*(24) = 2.795, *p* = .010. Bimodal: Trained Syllable, *t*(24) = 2.731, *p* = .012; Novel Syllable, *t*(24) = 3.158, *p* = .004; Trained Gender, *t*(24) = 2.681, *p* = .013; Novel Gender, *t*(24) = 2.840, *p* = .009).

### Discussion

The same pattern of results was obtained here as in the previous experiment: there was a main effect of Session—which could reflect a practice effect—and a main effect of Test Gender, which shows that participants did better on the Novel Gender (i.e., Female stimuli) items compared to the Trained Gender (i.e., Male stimuli) items. Additionally, here but not in Experiment 1A, there was a significant interaction of Test Gender by Test Syllable, which suggests that Female /na/ test items were easier than Male /na/ test items. Crucially, despite only training the participants on the most difficult minimal pair (Male /na33/-/na241/) and increasing the number of participants in each Distribution Condition, there was no difference between Unimodal and Bimodal distribution conditions at Posttest relative to Pretest. This suggests that there was no distributional learning effect since both groups improved significantly from Pretest to Posttest, which, as discussed in Experiment 1A, may simply reflect a general practice effect.

The results are in line with previous research [25,26], in which the participants were tested on a minimal pair of Mandarin lexical tones that was easy to discriminate and which also found no distributional learning of lexical tones. Taken together, the lack of significant distributional learning of lexical tones appears not to be due to the difficulty of the lexical tone minimal pair or to the particular target language. It appears that non-tone language adult learners do not acquire tones distributionally—at least not when the training phase involves passive listening. It may be the case that because the female stimuli were easy to discriminate even at Pretest, it may have bootstrapped the participants to discriminate the other minimal pairs. Another possibility relates to the participants’ attention to the training tokens. We suspect that the null results may be due to individual differences in their ability to sustain attention throughout the entire five-minute training phase. Would learners show an effect of distributional learning if they were prompted to listen attentively throughout the training set of stimuli? This is examined in Experiment 2.

## Experiment 2: Attended Listening

In Experiment 2, we repeat Experiment 1B with one crucial difference: modelled on electrophysiological studies [32,39], we added a control task during the training phase, in which pure tones were randomly inserted among the training tokens and participants were instructed to indicate on a paper response sheet when they hear a ‘beep’. This demands participants to pay attention to each sound heard during the training phase. Indeed, participants allocate more attentional resources on an attended listening task like this, as indexed by the presence of an ERP response (processing negativity, PN) [40], compared to a passive listening task [39].

### Method

#### Participants

Participants were 50 native Australian English listeners (42 females), who had not been tested in Experiment 1A or 1B. Their ages ranged between 17 and 40 years, with an average of 20.82 (SD_age_ = 5.32). None of the participants spoke a tone language and all reported normal hearing. Nineteen participants reported having minimal musical training (≤ 0.5 year = 2; 1 year = 11; 2 years = 6).

#### Ethics Statement

The University of Western Sydney Human Research Ethics Committee approved the study protocol. All participants were recruited from the University of Western Sydney. Participants were given an information sheet and they provided written informed consent prior to participating in the experiment. Written informed consent was obtained directly from the handful of participants who were 17-year-old undergraduates as per the protocol approved by the University of Western Sydney Human Research Ethics Committee (in Australia, it is common for 17 (going on 18) year olds to begin their first year of undergraduate degree). Participants were given course credit for their participation.

#### Stimuli

The same stimuli from Experiment 1A were used. In addition, the sine wave tone used in the practice task was also used as the beep tone during training.

#### Procedure

The participants were randomly assigned to one of two Distribution conditions: Unimodal or Bimodal. The procedure of Experiment 2 was similar to that of Experiment 1B except that the participants were required to complete an additional vigilance task during training. Participants were provided with a response sheet containing the numbers 1 to 288. They were instructed that they would hear a total of 288 sounds during this phase and that some of those sounds would be beeps occurring randomly throughout the sequence of sounds. The participants were told to follow the sound number being played and circle the sound number every time they hear a beep. A total of 32 beeps occurred interspersed randomly within 256 training tokens.

### Results

**Fig 5:**
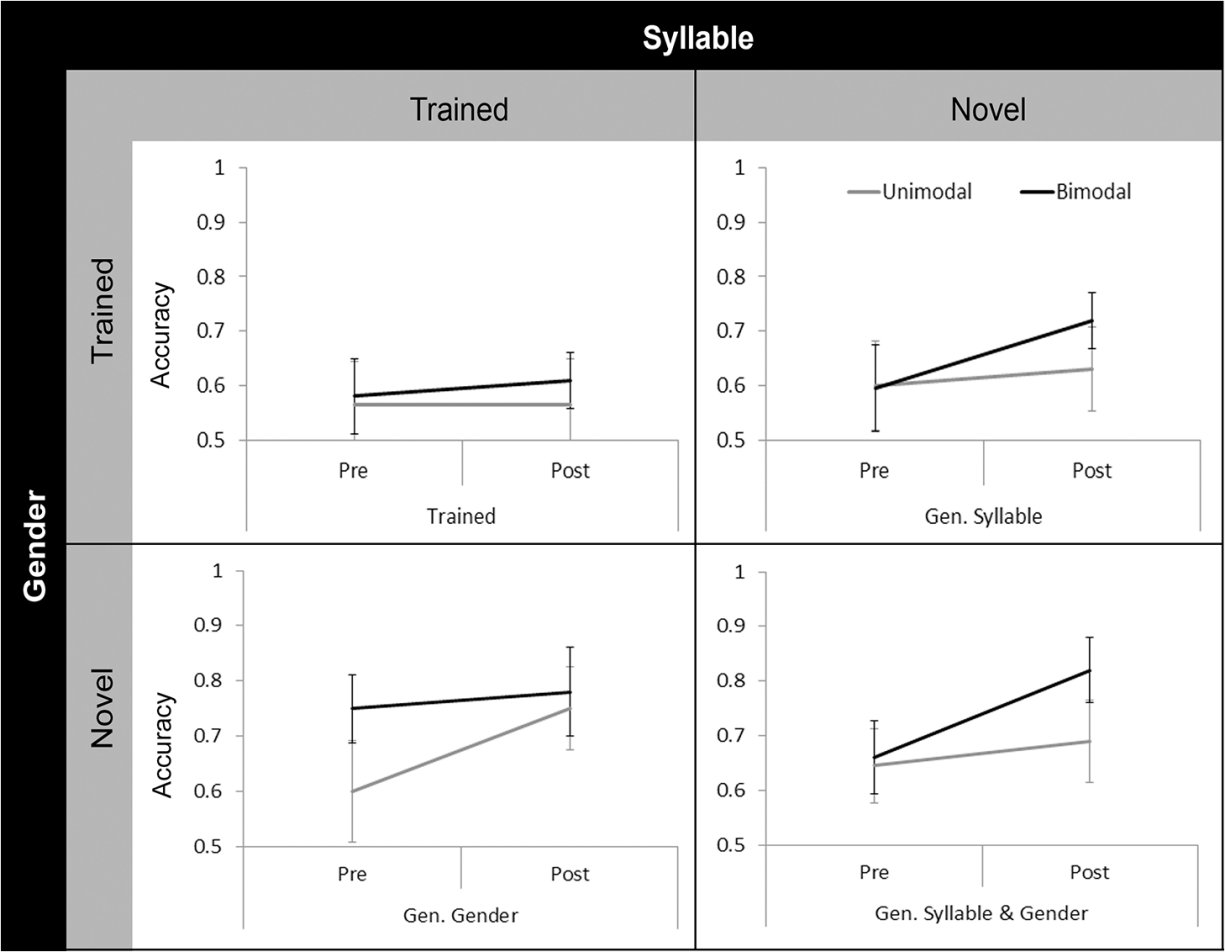
Comparison of Pretest and Posttest scores by Distribution Condition in Experiment 2. Just as in Experiment 1B, Trained, Gen. Syllable, Gen. Gender and Gen. Syllable & Gender test items represent scores on Male /na/, Male /k^h^a/, Female /na/ and Female /k^h^a/ tets items, respectively. In general, Posttest scores were higher than Pretest scores. The Bimodal group outperformed the Unimodal group on Novel Syllable test aspect at Posttest relative to Pretest. Note that the Bimodal group’s Pretest score on Female /na/ (Gen. Gender) was relatively high compared to that of the Unimodal group. Error bars represent 95% confidence intervals.

All the participants successfully identified the beeps in the cover task, so no participants were excluded from data analysis. Fig 5 illustrates the Pretest and Posttest scores on each test item by Distribution Condition. A 2 x (2 x 2 x 2) Mixed ANOVA with Distribution Condition (Unimodal vs. Bimodal) as a Between Subjects factor; and as Within Subjects factors: Session (Pretest vs. Posttest), Test Syllable (Trained vs. Novel) and Test Gender (Trained vs. Novel) revealed that there were main effects of Session (*F*(1, 48) = 18.774, *p* < .001, η_P_^2^ = .281) and Test Gender (*F*(1, 48) = 39.663, *p* < .001, η_P_^2^ = .452). In general, Posttest scores (M = .696, SE = .016) were higher than Pretest scores (M = .624, SE = .016); and Novel Gender (i.e., Female stimuli; M = .712, SE = .017) were easier to discriminate than Trained Gender (i.e., Male stimuli; M = .608, SE = .016). There was also a main effect of Distribution Condition (*F*(1, 48) = 4.266, *p* = .044, η_P_^2^ = .082): the Bimodal group (M = .689, SE = .02) had higher scores than the Unimodal group (M = .631, SE = .02). While a two-way interaction of Session by Distribution Condition was not significant (*F*(1, 48) = .832, *p* = .366, η_P_^2^ = .017), a three-way interaction of Session by Distribution Condition by Test Syllable was (*F*(1, 48) = 6.472, *p* = .014, η_P_^2^ = .119): at Posttest relative to Pretest the difference between the Bimodal group (M = .770, SE = .027) was greater than the Unimodal group (M = .660, SE = .027) on Novel Syllable (/k^h^a/) test items, but the two groups did not differ on Trained Syllable (/na/) test items.

A set of one-sample *t*-tests on difference scores was conducted to determine whether both distribution conditions showed significant improvement at Posttest relative to Pretest. After Holm-Bonferroni correction was applied, for the Bimodal group, three of the four test aspects showed significant improvement (Novel Syllable, *t*(24) = 4.806, *p* < .001; Trained Gender, *t*(24) = 3.668, *p* = .001; Novel Gender, *t*(24) = 4.507, *p* < .001 vs. Trained Syllable, *t*(24) = 1.224, *p* = .233). In contrast, for the Unimodal group, only one test aspect (Novel Gender, *t*(24) = 2.847, *p* = .009 vs. Trained Syllable, *t*(24) = 1.964, *p* = .061; Novel Syllable, *t*(24) = 1.208, *p* = .239; Trained Gender, *t*(24) = .350, *p* = .730) showed above chance improvement.

### Discussion

Experiment 2 investigated whether non-tone language listeners are able to acquire lexical tone categories after listening to a distribution of a lexical tone minimal pair in an attentive task. It was found that the participants generally showed better discrimination performance at Posttest relative to Pretest and on Novel Gender (female) stimuli relative to Trained Gender (male) stimuli. Importantly, this time, there was an indication of successful distribution learning: at Posttest compared to Pretest, the Bimodal participants performed better than the Unimodal participants on Novel Syllable (/k^h^a/) test items. Unexpectedly, there was no difference between the two Distribution Conditions on Trained Syllable (/na/) test items. However, inspection of the Pretest scores on Trained Syllable test items showed relatively high performance by the Bimodal group on Female /na/ test items compared to the Unimodal group. This may also explain the unexpected main effect of Distribution Condition. Regardless, the results suggest that the Bimodal group showed better discrimination performance after training than the Unimodal group.

These results are substantiated by comparing the one-sample *t*-tests across Experiments 1B and 2. Recall that in Experiment 1B, both the Unimodal and Bimodal conditions showed improvement above chance at Posttest relative to Pretest on all test aspects. However, in Experiment 2, the Unimodal group only showed above chance improvement on one test aspect (Novel Gender) while the Bimodal group showed significant improvement at Posttest on three aspects (Novel Syllable, Trained Gender and Novel Gender). The lack of improvement for the Trained Syllable by the Bimodal group may be again due to the relatively high performance in response to Female /na/ even at Pretest. Nevertheless, the results of Experiment 2 suggest that, by and large, not only did the Bimodal group improve significantly after exposure to the training tokens, the Unimodal group did *not* improve, which, taken together, indicates an effect of distributional learning in Experiment 2 when both groups’ attention was focused (by means of the ‘beep’ task) on the structure of the distribution encountered.

Qualitatively, it appears that the distribution structure had more of an effect on the participants in an attentive task compared to a passive task. Across all three experiments, we found the distributional learning effect only in Experiment 2, in which an attentive task was used. Indeed, a direct comparison of difference scores from Experiments 1B and 2 using a 2 x 2 x (2 x 2) Mixed ANOVA with between-subjects factors Attention (Experiment 1B vs. Experiment 2) and Distribution Condition (Unimodal vs. Bimodal) and within-subjects factors Familiarity (Trained vs. Novel) and Test Aspect (Syllable vs. Gender) revealed a main effect of Familiarity (*F*(1, 96) = 4.368, *p* = .039, η_P_^2^ = .044) and a 4-way interaction, (*F*(1, 96) = 6.278, *p* = .014, η_P_^2^ = .061). Simple main-effects analysis revealed that for the Unimodal group, participants in Experiment 2 scored significantly less than those in Experiment 1B on Trained Gender test aspect (M_Difference_ = 10.5, SE = 4.911, *p* = .038) and marginally less on Novel Syllable (M_Difference_ = 9.00, SE = 4.725, *p* = .063) after Sidak correction, while for the Bimodal group, the participants in both experiments did not differ on any test aspects, which as discussed, may be due to the lack of improvement on the Female /na/ test items by the Bimodal group in Experiment 2. Nonetheless, when the results across both experiments are taken together, the negative effect of a unimodal distribution (i.e., a suppression of improvement) is evident when the training tokens are listened to attentively, but not when listened to passively.

## General Discussion

These studies were designed to add to the current distributional learning literature by examining whether: (i) non-tone language listeners are able to acquire lexical tone categories distributionally; and (ii) attention to the training task modulates the effect of distributional learning effect of lexical tones. The results of this series of experiments suggest that lexical tones may be learned distributionally, as shown by the Bimodal group exhibiting improved discrimination of the endpoints of a lexical tone continuum, while the Unimodal group showed no corresponding improvement, but only when learners are encouraged to pay attention to the tones during the training phase. When learners were trained passively on either a Bimodal or a Unimodal distribution of a lexical tone minimal pair (Experiments 1A and 1B), the two groups showed generally higher performance at Posttest than at Pretest, which is likely due to general practice effects with the test stimuli. On the other hand, when the participants were given a task that encouraged attention to the distribution (Experiment 2), not only was the Bimodal groups’ performance on Novel Syllable test items significantly higher than that of Unimodal participants, but the Bimodal group also showed significant improvement from Pretest to Posttest while the Unimodal group showed no such improvement. This suggests that the distributional learning occurs under the conditions in Experiment 2, but not in Experiments 1A and 1B.

Furthermore, comparing the difference scores between all three experiments reveals that a suppression of improvement (which is a part of the outcome of distributional learning) is evident when Unimodal participants were trained attentively. In Experiment 2, the Unimodal participants showed no improvement on three of the four test aspects (Trained and Novel Syllables and Trained Gender), while in Experiments 1A and 1B, the Unimodal participants improved significantly on most, if not all, test aspects. This improvement by the Unimodal group in Experiments 1A and 1B and suppression of improvement by the Unimodal group in Experiment 2 is a novel finding, given that no distributional learning studies that employed a pretest-training-posttest design also used a unimodal distribution (e.g. Escudero et al., 2011). The lack of Unimodal suppression on Novel Gender (i.e., female) test aspect in Experiment 2 suggests that the suppression effect may not be strong enough to overcome the relative salience of female stimuli compared to male stimuli. Indeed, the results from Experiments 1A, 1B and 2 suggest that female stimuli were easier to discriminate than male stimuli. It should be noted that this is not due to the fact that there were many more female participants who may be more adept at discriminating stimuli that share a similar pitch range as themselves. The same pattern of results was found amongst the male participants: even at Pretest, female test stimuli were easier to discriminate than male test stimuli (Experiment 1A: *t*(10) = 2.091, *p* = .026; Experiment 1B: *t*(10) = 2.262, *p* = .047; Experiment 2: *t*(7) = 7.099, *p* < .001). The lack of significant difference by the Bimodal group in Experiment 2 relative to the Bimodal group in Experiment 1B may be confounded by the relatively high performance by the former on the Female /na/ test items. Future work could investigate this further by ensuring an equal Pretest performance across distribution groups and experiments. Nonetheless, taken together, these results are in line with previous research suggesting that statistical learning is more effective when attention is given to the to-be-learned stimuli than when stimuli are processed passively [29,30].

Our findings suggest that attention to the training task alone is enough for learners to show an effect of distributional learning without the need to draw the learners’ attention to a specific acoustic cue as has been done in studies using enhanced distributions [31]. Since learners allocated more attentional resource to the auditory stimuli in an attended task than an unattended task [39], we argue that learners in an attended task are better able extract the statistics of the distribution. What is not known, however, is whether general attention in the acquisition phase would result in comparable learning to when learners’ attention to the specific acoustic cue is manipulated via an enhanced distribution and whether there is any additive effect of both types of attention manipulations. Future studies should address these research questions as they will add to our knowledge of understanding how humans acquire language. Indeed, the simultaneous use of both types of attention is seen in infant-directed speech (IDS): analogous to enhanced distributions in distributional learning research, hyperarticulation of vowels and tones in IDS is proposed to facilitate infants in acquiring phonetic and lexical tone categories by highlighting the acoustic differences in vocalic and tonal contrasts [41–44], while the use of increased pitch and larger pitch modulations are presumed to capture infants’ attention to the speech sounds in general [43,44], similar to the attended task during the training phase in Experiment 2.

Still, the question of why attention to the acquisition phase and/or attention to the specific acoustic cue is required for distributional learning of lexical tones and vowels, but not consonants, remains unanswered. It is noteworthy that lexical tones and vowels are perceived less categorically than consonants, and consequently, both lexical tones and vowels overlap and have greater acoustic variability in their production to an extent not evident in consonants [37,45–47]. Therefore, perhaps attention, either in the training phase in general or to the specific acoustic cue, may be necessary during acquisition for speech sounds that tend to be variable in nature.

These studies have only considered the acquisition of lexical tone categories (as indexed by a discrimination task on the endpoints of a continuum before and after training) and only by non-tone language speakers. A possible future direction, then, is to investigate whether learners perceive those lexical tones categorically following training by comparing their discrimination at various points on the continuum, that is, the endpoints vs. the within-category tokens. Furthermore, could our results generalise to non-linguistic pitch categories (e.g. musical pitch categories)? If so, then this would suggest that distributional learning may underlie the perceptual attunement of musical systems as well, which would be in line with the Shared Sound Category Learning Mechanism hypothesis [48]. In addition, would there be differential effects for populations who use pitch extensively (such as tone language listeners and musicians)? Given that these groups tend to outperform non-tone language non-musicians in discriminating and learning lexical tones [49–52], would a brief distributional training on a lexical tone minimal pair still provide an advantage for the ‘pitch experts’ above and beyond their extensive experience with pitch? Work is currently being undertaken in our laboratory to address these issues.

In sum, it was found that, contrary to other studies on distributional learning of lexical tone [25,26], non-tone language listeners *are* able to acquire lexical tones distributionally, but only when learners actively attend to the training stimuli. Nonetheless, this suggests that lexical tones have the same linguistic status as consonants and vowels, at least in terms of acquisition. The present study also adds to the growing distributional learning literature by providing a direct comparison between unattended and attended training; distributional learning effect is more readily observed when an attended task is used during training than when an unattended task is used, presumably because the learners are better able to extract the relevant statistics of the distribution. In order to fully understand distributional learning as a learning mechanism, further research is required to investigate whether the same mechanism extends from speech to the music domain; whether there are population differences (language background; musical background) in the effects of distributional learning; and the role of attention during acquisition in general and to specific acoustic cues of the auditory stimuli.
